# Assessing the Association between the Degree of Pain and Socioeconomic Status among Older Persons in Ghana

**DOI:** 10.5539/gjhs.v6n3p155

**Published:** 2014-03-06

**Authors:** Kwame Annin, Bahiru I. I. Saeed, Alfred Yawson, A. A. I. Musah, Emmanuel Nakua, Peter Agyei-Baffour, N. N. N. Nsowah-Nuamah

**Affiliations:** 1 Mathematics and Statistics Department, Kumasi Polytechnic, Kumasi, Ghana; 2 Department of Community Health, University of Ghana Medical School, College of Health Sciences, Korle-Bu, Accra, Ghana; 3 Department of Community Health, Kwame Nkrumah University of Science and Technology Medical School, College of Health Sciences, Ghana; 4 Paediatric Department, Komfo Anokye Teaching Hospital, Kumasi, Ghana; 5 Rector, Kumasi Polytechnic, Kumasi, Ghana; 6 Mathematics and Statistics Department, Tamale Polytechnic, Tamale, Ghana

**Keywords:** degree of pain, predisposing factors, enabling factors, prevalence, ageing Ghanaians

## Abstract

**Objectives:**

The current study sought to examine the association between the degree of pain and socioeconomic status among older male and female Ghanaians.

**Method:**

Data were drawn from the 2007–08 World Health Organization Global Ageing and Adult Health (SAGE) survey conducted in Ghana (Young adults=803, Adults=1689 and Older adults=2616). This includes bodily aches Ghanaians experienced in the last 30 days. Analyses of the association of pain with predisposing and enabling factors were carried out by means of ordinal logistic regression analysis.

**Results:**

In the age-adjusted model, pain was statistically significantly associated with the cohabitating group as its marginal effect suggests that respondents in that category were less likely to experience pain as related to the others in women.

**Conclusion:**

This study established that Ghanaian men go through more pain than their women counterparts. This article is premier to our knowledge to apply ordered logistic for the degree of pain.

## 1. Introduction

Pain is a major public health problem. For individual pain causes decreased quality of life, activity limitations, and reduced functional capacity (Mantyselka, Turunen, Ahonen, & Kumpusalo, 2003; Mantyselka et al., 2001; Smith et al., 2001). For society, pain is a considerable financial burden causing an increased use of health services and medical, sickness absence and early retirement (Elliott et al., 2003; Mantyselka et al., 2001, 2002). Workability is strongly affected by pain (Blyth et al.,2003), regardless of its cause or site. Loss of productivity is a substantial consequence of pain in the work life. According to Mantyselka et al. (2003) 35% of the Finnish population reported chronic pain. Much research has examined meaning in the form of appraisals of the threat value of pain, or individuals’ perceived ability to cope with the threat of pain (Sullivan et al.,2001). Discourses of justice and injustice appear inherent in the chronic pain experience (McParland, Hezseltine, Serpell, Eccleston, & Stenner, 2011). Individuals with chronic pain may ascribe external blame for those suffering (McParland & Whyte, 2008), which may increase the likelihood that pain is experienced with an elevated sense of injustice (Miller, 2001).

Meanwhile, perceived injustice in the chronic pain context has been operationally defined as an appraisal reflecting the severity and irreparability of pain-related loss, blame, and unfairness (Sullivan et al.,2008). Mounting evidence indicates that perceived injustice contributes to problematic outcomes associated with persistent musculoskeletal pain. This has led to greater pain severity, pain behavior, and mental health difficulties, reduced physical function, and prolonged work disability (Scott & Sullivan 2012; Scott, Trost, Milioto & Sullivan, 2013; Sullivan et al., 2008; Sullivan, Davidson, Garfinkel & Siriapaipant, 2009; Sullivan et al.,2009). Moreover, due to the social inequality in the society, perceived injustice highly predicts adverse pain outcomes even when controlling for other pain-related psychosocial constructs, such as pain catastrophizing and fear of movement (Rodero et al., 2012; Scott & Sullivan, 2012; Sullivan, Davidson, Garfinkel, Siriapaipant & Scott, 2009; Sullivan et al.,2009). There are indications that perceived injustice might be more resistant to change than other psychosocial pain-related variables (Sullivan et al.,2008).

However, other recent studies argued that peripheral and central sensitization in knee osteoarthritis (OA) could be important for the poor pain outcome for some patients after total knee arthroplasty (TKA) and pharmacological interventions (Arendt-Nielsen & Graven-Nielsen, 2011; Skou et al., 2012). Quantitative sensory testing (QST) has frequently been applied to investigate sensitization in OA and increased pain sensitivity both locally and distantly from the affected joint has been reported (Arendt-Nielsen et al., 2010; Graven-Nielsen, Wodehouse, Langford, Arendt-Nielsen & Kidd, 2012; Imamura et al., 2008; Lee et al., 2011; Skou et al., 2013; Suokas et al.,2012). In patients with chronic painful knee OA, higher clinical pain intensities and longer pain durations caused relatively more temporal summation of pain compared with patients with shorter duration and less pain (Arendt-Nielsen et al.,2010).

Moreover, chronic pain and co-morbid insomnia are worldwide recognized as serious health problems that severely impact patients’ quality of life and productivity. Sleep disturbances are acknowledged among patients with nociceptive pain (Abad, Sarinas & Guilleminault, 2008; Irwin et al., 2012; Smith, Quartana, Okonkwo & Nasir, 2009; Taylor-Gjevre, Gjevre, Nair, Skomro & Lim, 2011), neuropathic pain (Langley, Van Litsenburg, Cappelleri, Carroll, 2013; Zelman, Brandenburg & Gore, 2006) and mixed pain conditions such as cancer (Buffum et al., 2011; Cheng & Yeung, 2013; Dhruva et al., 2012; Garrett et al.,2011) or low back pain (Alsaadi, McAuley, Hush & Maher, 2011; Bahouq, Allali, Rkain, Hmamouchi & Hajjaj-Hassouni, 2012; Marty et al., 2008; van de Water, Eadie & Hurley, 2011). Nevertheless, it is imperative if treatments of chronic pain are to be successful that chronic pain is understood. Failure to do so causes many to suffer and modeling this could prove vital for research in this area. To date, however, socioeconomic and socio-demographic predictors have not been empirically researched into in Ghana. Against this backdrop, the study seeks to examine the association between socioeconomic status and the degree of pain among older male and female Ghanaians.

## 2. Material and Methods

### 2.1 Sampling Procedures

The data employed in this study were drawn from the World Health Organization Global Ageing and Adult Health (SAGE). This aims to evaluate the association of the degree of pain and predisposing and enabling factors of adults and ageing Ghanaians. It also aims at addressing the gap in reliable data and scientific knowledge on ageing and health in low – and middle –income countries. SAGE is a longitudinal study with nationally representative samples of persons aged 50+ years in Ghana with a smaller sample of adults aged 18-49 years. Instruments are compatible with other large high-income country longitudinal ageing studies. Wave 1 was conducted during 2007-2008 and included a total of 4305 respondents aged 50+ and 803 aged 18-49. In this article, Sample sizes of young adults=803, adults=1689 and older adults=2616 respectively had been considered.

Multistage cluster sampling strategies were used where households were classified into one of two mutually exclusive categories:

(1) All persons aged 50 years and older were selected from households classified as ‘50+ households’; and
(2) One person aged 18–49 years were selected from a household classified as an ‘18–49 household’.

Household enumerations were carried out in the final sampling units. One household questionnaire was completed per household where a household informant and individual respondent need not be the same individual. One individual was selected from 18–49 households, whereas for 50+ households all individuals aged 50+ were invited to complete the individual interview. Proxy respondents were identified for selected individuals who were unable to complete the interview. Household-level analysis weights and person-level analysis weights were calculated for each country, which included sample selection and a post-stratification factor. Post stratification correction techniques used the most recent population estimates provided by the Ghana Statistical Service (Biritwum et al.,2013).

### 2.2 Measures of Pain

Information about pain status was elicited in one question. Respondents were asked the degree of bodily aches or pains they experienced in the last thirty days. The question captures the degree of pain. Body aches or pains refer to any form of physical pain or discomfort in the body that interferes with a person’s usual activities, either for a short or long period of time.

### 2.3 Predisposing and Enabling Measures

Predisposing and enabling measures were selected on the basis of previous studies on the prevalence of acute, chronic and disabling pain (Saastamoinen et al.,2005).

Predisposing measures were age (age 18-49 (young adults), 50-59 (adults) and 60yrs and above (older adults)), gender and marital status (currently married, never married, cohabitating, separated/divorced and widowed).

Enabling measures were assessed in terms of education, job employment, well-being and income. Education was recorded as *college/university completed, high school completed, secondary school completed, primary school completed, less than primary school completed and no formal education*. Job employment was categorized into four groups: public, private, self-employed and informal employment. Public sector includes employees of state, or municipal governments and their agencies, parastatal enterprises, and semi-autonomous institutions such as social security institutions that are owned by the government or institutions like religious schools if the staff are paid by the government. Private sector includes any employees not working for the government and not self-employed. Self-employed includes those who earn their livelihood directly from their own trade or business rather than as an employee of another. Informal employment could mean employment in the informal economy or informal employment. Informal economy refers to the general market income category wherein certain types of income and the means of their generation are “unregulated by the institutions of society, in a legal and social environment in which similar activities are regulated”. Jobs in the informal economy are characteristically without benefits such as health insurance, sick leave, paid vacations or pensions. Well-being status was recorded as *completely, mostly, moderately, a little and not at all*. Income level was divided into five categories: Income Quintile; *Q1 (lowest) through Q5 (highest)*. Wealth or income Quintiles were derived from the household ownership of durable goods, dwelling characteristics (type of floors, wells and cooking stove), and access to services (improved water, sanitation and cooking fuel) for a total of 21 assets. A two-step random effects probit model was used to generate the Quintiles (Kowal et al.,2012).

### 2.4 Statistical Methods

Descriptive characteristics of all respondents were first calculated. Analyses of the association of pain with predisposing and enabling factors were carried out separately for women and men by means of ordinal logistic regression analysis. Odds ratios (OR) and their respective 95% confidence intervals were also computed. Firstly, no variable was adjusted for age (age-unadjusted model). Secondly, each variable in the analysis was adjusted for age (age-adjusted model).

For a response with five options (extreme, severe, moderate, mild and none), the ordinal logistic regression suggests that the effect of any predisposing and enabling factors would induce a change in the odds of responding to option extreme instead of the rest, or extreme or severe or moderate or mild instead of none by a factor of the exponent of the regression estimates. Stata SE (version 12.1) was used for analysis.

## 3. Results

### 3.1 Ratio of Pain by Predisposing Factors

Age gradient was found in the degree of pain: the older the respondent, the higher the prevalence of pain (Table 1). By marital status, degree of pain varied only modestly in men and widely in women (Table 2).

**Table 1:** Descriptive characteristics of pain by age

Age Category	Male	Female	Sample size(n)
	Pain %	Pain %	
18 - 49	16.1	14.8	803
50 - 59	35.6	31.0	1689
60 +	48.3	54.2	2616

**Table 2:** Descriptive characteristics of pain by socio-demographic and socio- economic indicators

	Male	Female	Both Sexes
	Pain %	Pain %	Pain %
**MARITAL STATUS**			
Currently Married	83.1	31.5	58.9
Never married	2.4	2.5	2.5
cohabitating	1.2	1.4	1.3
Separated / divorced	7.5	19.9	13.3
Widowed	5.9	44.6	24.1
**HIGHEST EDUCATIONAL LEVEL**			
No formal education	38.7	59.3	48.4
Less than primary school	10.6	13.3	11.9
Primary school completed	14.2	9.9	12.2
Secondary school (O&A levels) completed	8.5	3.6	6.2
High school (or equivalent) completed	23.2	11.9	17.9
College / university completed	4.8	2.0	3.5
**JOB EMPLOYMENT**			
Public	13.3	5.0	9.4
Private	6.0	2.1	4.2
Self-employed	73.8	86.3	79.7
Informal employment	6.9	6.6	6.8
**INCOME**			
Q1	18.1	20.2	19.1
Q2	18.8	20.3	19.5
Q3	19.7	20.4	20.0
Q4	20.9	20.6	20.8
Q5	22.5	18.5	20.6
WELL-BEING			
Completely	1.7	1.1	1.4
Mostly	5.6	5.4	5.5
Moderately	25.6	21.6	23.7
A Little	43.0	43.7	43.4
Not at all	24.1	28.2	26.0

### 3.2 Ratio of Pain by Enabling Factors

In women, the degree of pain varied widely with educational levels (Table 2). Generally, there is decline of the pain ratio with education when estimating the ratios for both sexes. An educational gradient was found for pain (except high school completed) with lower level of education, having a greater percentage.

Self-employed Ghanaians tended to experience the greatest degree of pain in men and women respectively (Table 2). There was no income gradient found in the degree of pain in both men and women respectively. However, the well-being gradient was found for pain: the lower the well-being, the sharp intensity of pain.

### 3.3 Associations between Levels of Pain Outcomes and Predisposing and Enabling Factors among Men

Among men, pain showed age-unadjusted association with marital status. The marginal effect of being widowed suggests that respondents in that group were slightly more likely to experience pain than the rest. In contrast, the same cannot be said after adjusting for age. In age-unadjusted model, pain was associated with level of education. The marginal effects of having primary, secondary or high school education clearly suggests that respondents in those groups were less likely to experience various degrees of pain as compared to the others. After adjustment for age, the only remaining statistically significant association as the marginal effect of high school education suggest that respondents in that class were slightly less likely to experience pain as compared to the other groups. Pain showed age-unadjusted association with lower employment class among men as the marginal effect of being in the informal sector suggest Ghanaians in that class decreased the likelihood to experience pain as their other counterparts. There was no association at all after age-adjustment. Among men, the degree of pain was statistically significantly associated with the well-being in both the age-unadjusted and age-adjusted models (Table 3). In the age-unadjusted model, the various marginal effects on the well-being clearly suggest that, Ghanaians in those classes were more likely to have various degrees of pain than their counterparts with the complete well-being. These statistically significant associations remained unchanged after adjusting for age.

**Table 3:** Age-adjusted and age unadjusted odds ratio (OR) from ordinal logistic regression analysis and their 95% confidence intervals (CI) for the degree of pain by socio-demographic and socioeconomic indicators among males, females and altogether

	MALE				FEMALE				BOTH			
	Age Unadjusted		Age Adjusted		Age Unadjusted		Age Adjusted		Age Unadjusted		Age Adjusted	
	OR	CI	OR	CI	OR	CI	OR	CI	OR	CI	OR	CI
**AGE**																							
**18-49**	ref		ref		ref		ref					
50- 59			2.27 (1.80, 2.85 )				2.79 (2.13, 3.65)				2.51(2.11, 2.98)	
60 +			4.56 (3.62, 5.75)				4.52 (3.40, 6.01)				4.58(3.83, 5.47)
**MARITAL STATUS**											
**Currently married**	ref		ref		ref		ref					
Never married	0.85 (0.52, 1.37 )		1.38 (0.85, 2.23)		1.19 (0.71, 1.98 )		1.49 (0.87, 2.53)		0.96 (0.67, 1.35)		1.40 (0.98, 2.00)	
cohabitating	1.03 (0.53, 1.98)		1.12 (0.57, 2.15)		0.23 (0.10, 0.50)		0.30 (0.13, 0.65)		0.51 (0.31, 0.83)		0.60 (0.36, 0.99)	
Separated / divorced	0.94 (0.72, 1.23)		0.78 (0.59, 1.02)		1.42 (1.14, 1.77)		1.08(0.86, 1.35)		1.20 (1.02, 1.42)		0.95 (0.81, 1.13)	
Widowed	1.40 (1.04, 1.89)		1.07 (0.79, 1.45)		1.64 (1.37, 1.96)		1.10 (0.91, 1.34)		1.53 (1.32, 1.77)		1.05 (0.90, 1.22)	
**HIGHEST EDUCATION**																							
**No formal education**	ref		ref		ref		ref						
Less than primary school	0.87 (0.68, 1.10)		1.01 (0.79, 1.29)		0.65 (0.51, 0.82)		0.80 (0.63, 1.01)		0.74 (0.63, 0.88)		0.90 (0.76, 1.07)	
Primary school completed	0.59 (0.47, 0.74)		0.80 (0.63, 1.01)		0.49 (0.38, 0.65)		0.66 (0.50, 0.87)		0.54 (0.45, 0.64)		0.73 (0.61, 0.87)	
Secondary school completed	0.74 (0.56, 0.99)		1.11 (0.83, 1.50)		0.53 (0.34, 0.83)		0.95 (0.60, 1.51)		0.66 (0.52, 0.83)		1.03 (0.81, 1.32)	
High school(or equivalent) completed	0.63 (0.51, 0.77)		0.8 (0.65, 0.98)		0.48 (0.37, 0.62)		0.67 (0.51, 0.88)		0.56 (0.48, 0.66)		0.75 (0.63, 0.88)	
College/university completed	0.77 (0.53, 1.12)		0.99 (0.67, 1.45)		0.48 (0.26, 0.86)		0.60 (0.33, 1.09)		0.64 (0.47, 0.88)		0.83 (0.60, 1.14)	
**JOB EMPLOYMENT**												
**Public**	ref		ref		ref		ref					
Private	0.75 (0.52, 1.07)		0.84 (0.58, 1.20)		0.50 (0.27, 0.94)		0.60 (0.32, 1.14)		0.68 (0.50, 0.92)		0.77 (0.56, 1.05)	
Self-employed	1.05 (0.84, 1.32)		1.20 (0.95, 1.51)		0.86 (0.59, 1.24)		0.95 (0.65, 1.38)		0.98 (0.81, 1.19)		1.12 (0.92, 1.36)	
Informal employment	0.63 (0.45, 0.89)		0.72 (0.50, 1.01)		0.77 (0.49, 1.23)		0.84 (0.52, 1.34)		0.72 (0.55, 0.94)		0.82 (0.62, 1.07)	
**WELL-BEING**												
**Completely**												
Mostly	1.06 (0.56, 2.04)		1.04 (0.54, 2.04)		0.53 (0.23, 1.19)		0.60 (0.27, 1.35)		0.84 (0.51, 1.39)		0.87 (0.52, 1.45)	
Moderately	1.98 (1.11, 3.61)		1.96 (1.08, 3.61)		0.97 (0.45, 2.09)		1.01 (0.48, 2.18)		1.54 (0.98, 2.46)		1.53 (0.96, 2.46)	
A little	2.54 (1.43, 4.60)		2.45 (1.36, 4.50)		1.17 (0.55, 2.50)		1.20 (0.57, 2.57)		1.90 (1.20, 3.01)		1.85 (1.17, 2.96)	
Not at all	2.82 (1.57, 5.17)		2.52 (1.38, 4.70)		1.66 (0.78, 3.58)		1.64 (0.77, 3.53)		2.37 (1.49, 3.79)		2.19(1.37, 3.52)	
**INCOME**												
**Q1**	ref		ref		ref		ref					
Q2	1.24 (0.99, 1.57)		1.27 (1.00, 1.60)		1.03 (0.81, 1.30)		0.98 (0.78, 1.24)		1.12 (0.95, 1.32)		1.10 (0.94, 1.30)	
Q3	1.24 (0.98, 1.56)		1.12 (0.89, 1.42)		1.29 (1.02, 1.63)		1.26 (0.99, 1.59)		1.26 (1.07, 1.48)		1.18 (1.00, 1.40)	
Q4	1.09 (0.86, 1.39)		1.00 (0.78, 1.27)		0.97 (0.76, 1.24)		0.89 (0.69, 1.14)		1.04 (0.87, 1.23)		0.94 (0.79, 1.12)	
Q5	0.93 (0.72, 1.20)		0.79 (0.61, 1.03)		1.19 (0.91, 1.56)		1.07 (0.81, 1.40)		1.02 (0.85, 1.23)		0.89 (0.74, 1.08)	

Interestingly, in the age-adjusted model, age remained statistically significantly associated with pain and the magnitude of the associations were higher than that of the well-being. The marginal effect of age suggests that adults and older adults were more likely to experience degrees of pain than young adult in the age-adjusted model.

Among men, the degree of pain showed both age-unadjusted and age-adjusted associations with lower income. The marginal effect of being in the second income quintile, suggests that Ghanaians in that class were slightly more likely to experience pain than the other groups.

### 3.4 Associations between Pain Outcomes and Predisposing and Enabling Factors among Women

In comparison to men, age showed statistically significantly associated with pain. The marginal effect of age suggests that, adults and older adults were more likely to experience pain than young adult in the age-adjusted model.

In contrast to men, pain among women was also associated with marital status, as the various marginal effects of separated/divorced or widowed suggest respondents in those groups were slightly more likely to experience pain as those currently married.

After adjustment for age, the only remaining statistically significant association as the marginal effect of cohabitating suggests Ghanaians in that category were far less likely to experience pain as compared to the rest. Again, in contrast to men, the pain was associated with many more levels of education in the age-unadjusted model. The marginal effects of those levels of education, suggest that Ghanaians in these levels of education were fairly less likely to experience various degrees of pain as to their counterparts with no formal education. However, after adjustment for age, the only remaining statistically significant association as the marginal effect of primary and high school education suggest that respondents in those categories were fairly less likely to experience pain as compared to the others. In contrast to men, pain showed age-unadjusted association with rather higher employment class as the marginal effect of being in the private sector suggest respondents in that class decreased the likelihood to experience pain as the other groups.. After adjusting for age, the association did not exist.

Finally, in contrast to men, pain showed age-unadjusted association with income as the marginal effect of being in the third income quintile slightly increased the likelihood to experience pain as compared to the other Quintiles. There was absolutely no association after age-adjustment.

## 4. Discussion

This study established that Ghanaian men go through more pain than their women counterparts. The prevalence of pain in men were higher in both young adults and adults respectively, whereas in the older adults, the sex gradient was the opposite. The separated/divorced percentage was almost thrice in women as compared to men due to pain. More than twice Ghanaian men were currently married than their women counterparts. According to previous results (McParland et al.,2011), discourses of justice and injustice appear inherent in the chronic pain experience. Interestingly, individuals with chronic pain may ascribe external blame for (McParland, & Whyte, 2008), which may increase the likelihood that pain is experienced with an elevated sense of injustice. The degree of pain poses serious threat to the general population

In this study, the prevalence of pain was fairly the same among women and men. This is in sharp contrast to Saastamoinen et al. (2005). In educational levels, the degree of pain varied a bit wider in women than men. There was no employment gradient found in the degree of pain. However self-employed Ghanaians tended to experience the greatest degree of pain. This was supported by Blyth et al. (2003) as they claimed that work ability was strongly affected by pain regardless of its cause. There was an inverse relationship between the well-being and the intensity of pain. This was corroborated by Mantyselka et al. (2003 & 2001) and Elliott et al. (2001) as they established that individual pain causes decreased quality of life, activity limitations and reduced functional capacity. Loss of productivity is a substantial consequence of pain in the work life. In further disagreement with our results (Elliott et al., 2003; Mantyselka et al., 2001, 2002) maintained that pain has a considerable financial burden due to an increased use of health services and medical. Perceived injustice has been associated with greater pain severity, pain behavior, and mental health difficulties, reduced physical function, and prolonged work disability (Scott & Sullivan, 2012; Scott et al., 2013; Sullivan et al., 2008; Sullivan et al.,2009) similarly the marginal effect of widowed Ghanaians suggests they were slightly more likely to experience pain than the rest (age-adjusted model). Studies with other indicators also suggest adverse pain outcomes even when controlling for other pain related psychosocial constructs, such as pain catastrophizing and fear of movement (Rodero et al., 2012; Scott & Sullivan, 2012; Sullivan et al.,2009). The low pain association was consistent with education among men and women in the age-unadjusted model. However, in the age-adjusted model, it was associated fairly among women and weakly among men. The underlying assumption of the low prevalence of pain among those with various levels of education may lie in the small gap in resources provided by education. Weak resources only aid exposures, lack of care and chronic symptoms. However, previous studies (Saastamoinen et al.,2005) suggested pain association being most consistent with education among women. Sleep disturbances are acknowledged among patients with nociceptive pain (Abad et al., 2008; Irwin et al., 2012; Smith et al., 2009; Taylor-Gjevre et al.,2011), neuropathic pain (Langley, Van Litsenburg, Cappelleri, Carroll, 2013; Zelman, Brandenburg & Gore, 2006) and mixed pain conditions such as cancer (Buffum et al., 2011; Cheng & Yeung, 2013; Dhruva et al., 2012; Garrett et al.,2011) or low back pain (Alsaadi et al., 2011; Bahouq et al., 2012; Marty et al., 2008; van de Water et al.,2011). In our study, pain was highly associated with Ghanaian men in middle and lower classes. This could be attributed to the overburden of relations outside the nuclear families and also low incomes earned. Social (well-being) class has rarely been used in researches of pain. However, some researches have established that chronic pain and co-morbid insomnia are worldwide recognized as serious health problems that severely impact a patient’s quality of life and productivity. In this article, pain associated with employment was low in both sexes for the age-unadjusted and non-existent in the age-adjusted model. Nevertheless, exposures at work are likely to vary between social classes. Pain, among those in informal employment might be more pronounced than among those in public employment. Arguably, one would have expected stronger correlations between pain and job employment than pain and education since the data used consist of ageing Ghanaians who often have long-lasting work-related exposures. Income correlation with pain was found in the relatively low income bracket (Q2) in the age-adjusted model for men. However, the relationship was found in the middle income bracket (Q3) in the age-unadjusted model for women. According to literature, housing tenure has been associated with pain (Smith et al.,2001). Nevertheless in a longitudinal research, similar disparities could not be established (Elliot et al., 2002).

This research investigated the degree of pain as a problem. The data used, was cross-sectional and therefore relied heavily on self-reports. Pain is a subjective phenomenon and thus self-reports provided reliable data for the assessment of pain in ageing Ghanaians. The response rate of the survey (81%) conforms with past surveys on pain (response rate 67%) (Saastamoinen et al.,2005).

In conclusion, this article is premier to our knowledge and has thus found predisposing and enabling factor disparities in the degree of pain. Older adults were at the greatest risk for the degree of pain. Those with no formal education and self-employed were at the greatest risk for the degree of pain. Furthermore, Ghanaians in lower social (well-being) class were at greater risk of pain. Enabling factor disparities were at variance as pain result was severer. For this article, older adults with no formal education and self-employed are at risk of higher degree of pain. Further research is required to improve the enabling factor disparities in pain.
